# Effects of GABA_B_ receptors in the insula on recognition memory observed with intellicage

**DOI:** 10.1186/s12993-017-0125-4

**Published:** 2017-04-17

**Authors:** Nan Wu, Feng Wang, Zhe Jin, Zhen Zhang, Lian-Kun Wang, Chun Zhang, Tao Sun

**Affiliations:** 1 Ningxia Key Laboratory of Cerebrocranial Disease, Incubation Base of National Key Laboratory, Ningxia Medical University, Yinchuan, Ningxia China; 2 Department of Neurosurgery, General Hospital of Ningxia Medical University, Yinchuan, Ningxia China; 3 Department of Neuroscience, Uppsala University, Uppsala, Sweden

**Keywords:** GABA_B_R, Insula, Recognition of memory, Intellicage

## Abstract

**Background:**

Insular function has gradually become a topic of intense study in cognitive research. Recognition memory is a commonly studied type of memory in memory research. GABA_B_R has been shown to be closely related to memory formation. In the present study, we used intellicage, which is a new intelligent behavioural test system, and a bilateral drug microinjection technique to inject into the bilateral insula, to examine the relationship between GABA_B_R and recognition memory.

**Methods:**

Male Sprague–Dawley rats were randomly divided into control, Sham, Nacl, baclofen and CGP35348 groups. Different testing procedures were employed using intellicage to detect changes in rat recognition memory. The expression of GABA_B_R (GB1, GB2) in the insula of rats was determined by immunofluorescence and western blotting at the protein level. In addition, the expression of GABA_B_R (GB_1_, GB_2_) was detected by RT-PCR at the mRNA level.

**Results:**

The results of the intellicage test showed that recognition memory was impaired in terms of position learning, punitive learning and punitive reversal learning by using baclofen and CGP35348. In position reversal learning, no significant differences were found in terms of cognitive memory ability between the control groups and the CGP and baclofen groups. Immunofluorescence data showed GABA_B_R (GB1, GB2) expression in the insula, while data from RT-PCR and western blot analysis demonstrated that the relative expression of GB1 and GB2 was significantly increased in the baclofen group compared with the control groups. In the CGP35348 group, the expression of GB1 and GB2 was significantly decreased, but there was no significant difference in GB1 or GB2 expression in the control groups.

**Conclusions:**

GABA_B_R expression in the insula plays an important role in the formation of recognition memory in rats.

## Background

The insula in humans is located in the deep side of the lateral fissure, which is also known as the “hidden fifth lobe”. The position of the insula is deep, the surrounding structure is complex, and there is contact with the vast majority of brain areas [1]. In recent years, with advances in functional imaging and the development of deep brain electrical technology, the function of the insula has received substantially more attention. Recent studies have suggested that the insular cortex is the key node of the brain salience network (Salience network) [2]. The insula integrates body perception, produces subjective feelings, determines stimulus-driven attentional capture, coordinates neural resources, and causes the body to respond to stimuli. Additionally, studies have found that the insula of rodents plays a central role in the formation of taste and visual recognition memory [3, 4], and the anterior insula is an important area for perception and arousal in humans [5].

Recognition memory is a subcategory of declarative memory, which is an important index with which to evaluate the level of memory consolidation. Recognition memory includes at least two different memory processes: recollection and familiarity [6]. Recognition memory depends on many memory sub-systems of the brain network, including the visual pathway, temporal lobe medial structure (hippocampus and olfactory cortex) [4], frontal lobe and parietal cortex. Moreover, different brain regions are interrelated, highly integrated, and play different roles in recognition memory [7]. At present, many studies have focused on the role of the medial temporal lobe structure and frontal cortex in recognition memory. Although the structure of the insula has been studied, most studies utilized fMRI (functional magnetic resonance imaging) [5, 8]. The use of stereotactic microinjection technology to study the relationship between the insula and recognition memory is still rare [1].

GABA_B_R is a metabotropic receptor of GABA that mediates slow and sustained inhibitory effects. GABA_B_R is composed of two subunits, GB1 and GB2. GABA plays a role in relieving stress and calming the excitement of nerves by acting on GABA_B_R. When GABA binds to GABA_B_R, the G protein is activated. Then, a reduction in the presynaptic Ca^2+^ influx and inhibition of the release of a neurotransmitter or an increase in postsynaptic membrane K^+^ efflux leads to posterior membrane hyperpolarization and has an effect on G protein activation [9]. Post-synaptic, GABA_B_R activation can also enhance GABA_A_R function that is outside of the synapse to maintain normal network function [10]. Recent studies have shown that GABA_B_R participates in many important physiological activities and pathological changes. GABA_B_R directly interacts with transcription factors, regulates long-term protein synthesis and metabolic activities, plays a central role in hippocampal neuron hyperactivity [11] and is important for memory consolidation [10, 12]. Moreover, GABA_B_R is a key regulator of neurogenesis, synaptic plasticity and the long-term potentiation (LTP), which are important for guiding the regulation of long-term memory [12–14]. The GABA_B_R agonist baclofen impairs learning and memory, while the GABA_B_R antagonist CGP35348 improves cognitive processing. Baclofen inhibits spatial learning in mice by activating the TREK-2K^+^ channel through the PKA pathway [15]; it also promotes the disappearance of normal elastic memory traces and disrupts the consolidation of conditioned reward memory [16]. Although the contribution of GABA_B_R to memory has been widely recognized, some contradictory conclusions exist due to differences in behavioural tasks, animal strains, gender, drug concentrations, time, and pathways used in experimental animals, so the relationship between the regulation of learning and memory and GABA_B_R expression still warrants further research [14, 17]. Furthermore, the role and mechanism of GABA_B_R in the recognition of the insula is not yet clearly understood.

In the present study, we focused on cognitive function in normal rats after treatment with the GABA_B_ receptor agonist baclofen and the antagonist CGP35348, which were injected into the bilateral insula. Furthermore, by controlling the changes in GABA_B_R expression in the insula, we examined the behavioural changes in recognition memory via intellicage.

## Methods

### Reagents

Primary antibodies against GB_1_R and GB_2_R were purchased from Abcam (Cambridge, UK). GABA_B_ receptor agonist Baclofen and antagonist CGP35348 were obtained from Sigma (St. Louis, US). The First Strand cDNA Synthesis Kit was purchased from Thermo Fisher. The PCR primers were designed and synthesized by Sangon Biotech (Shanghai, CN). The BCA Protein Assay Kit and Total Protein Extraction Kit were purchased from Jiangsu KeyGEN BioTECH Corp, Ltd.

### Animals

Male Sprague–Dawley rats (6–8 weeks old, 250–300 g) were provided from the Animal Center of Ningxia Medical University. Each rat was singly housed with an alternating 12:12 h light/dark cycle. These rats were randomly divided into a control group (Control), Sham operation group (Sham), saline group (Nacl), Baclofen group (BLF), and CGP 35348 group (CGP), (sham group was similar in operation with saline group, but was injected with drugs and saline), with five animals in each group. All animal use procedures were approved by the Ningxia Medical University Medical Center Animal Care and Use Committee and were conducted in accordance with the National Institutes of Health Guide for the Care and Use of Laboratory Animals. (No. 2016-124).

### Model establishment

After an acclimatization period of at least 1 week, the animals received surgical implantation of cannulae aimed at the insular cortex according to a standardized protocol [18].

### Surgery

Rats were implanted with bilateral canulas aimed at the granular insular cortex. Before surgery, animals were anesthetized with 10% chloral (4 ml/kg, ip.). The animals were mounted into a stereotaxic frame used to position the 22-gauge stainless steel guide canula in the granular insulars. Coordinates obtained from the Paxinos and Watson brain atlas (mm from bregma: AP = +1.2; ML = ±5.5; mm from skull surface: DV = −6.5). The guide canula was anchored to the skull using stainless steel screws and acrylic cement [19]. The animals were allowed 14 days for recovery after guide canula surgeries before the behavioural test.

### Microinjection procedure

All microinjections were done slowly (1 µl/0.5 min) using a 5 µl Hamilton syringe connected by Pe-20 polyethylene tube. The stainless steel injection needle (50 G) was cut to protrude 0.5 mm beyond the tips of the guide cannulae and left in place for 1 min after injection to allow diffusion of the solution and to prevent back flow. Saline (0.3 nmol/μl), Baclofen (125 ng/μl) [20], and CGP35348 (12.5 µg/µl) [21], were injected bilaterally into the granular insular 30 min before the start of the behavioural test each day.

### Implanted signal transponders

After 14 days recovery from guide canula surgeries and 24 h before introduction into intellicage, the rats were anesthetized with 10% chloral (4 ml/kg, ip.) and the transponders were implanted subcutaneously into the scapula of rats using the injector system delivered with the intellicage. The detector was then used to verify their appropriate subdermal location by read-out of the transponder [22].

### Intellicage

The intellicage (TSE Systems GmbH,Germany; http://www.newbehavior.com) is an automated group-housing apparatus allowing experimental testing within the home cage. The cage (410 × 190 × 435 cm) is equipped with four operant conditioning chambers located in each corner. Each conditioning chamber contains two water-drinking bottles and is accessible by a small opening containing a transponder reader antenna that registers the microchip of the entering rat. Access to each water bottle is controlled by gated nosepoke holes containing infrared beam-break sensors, which can be programmed to open or remain closed upon visit or nosepoke response. As each rat was implanted with a unique microchip, corner entry and nosepoke data could be integrated with microchip readings collected by each conditioning corner’s antenna, allowing data to be separated by each individual rat.

### Behavioural test

**Fig. 1:**
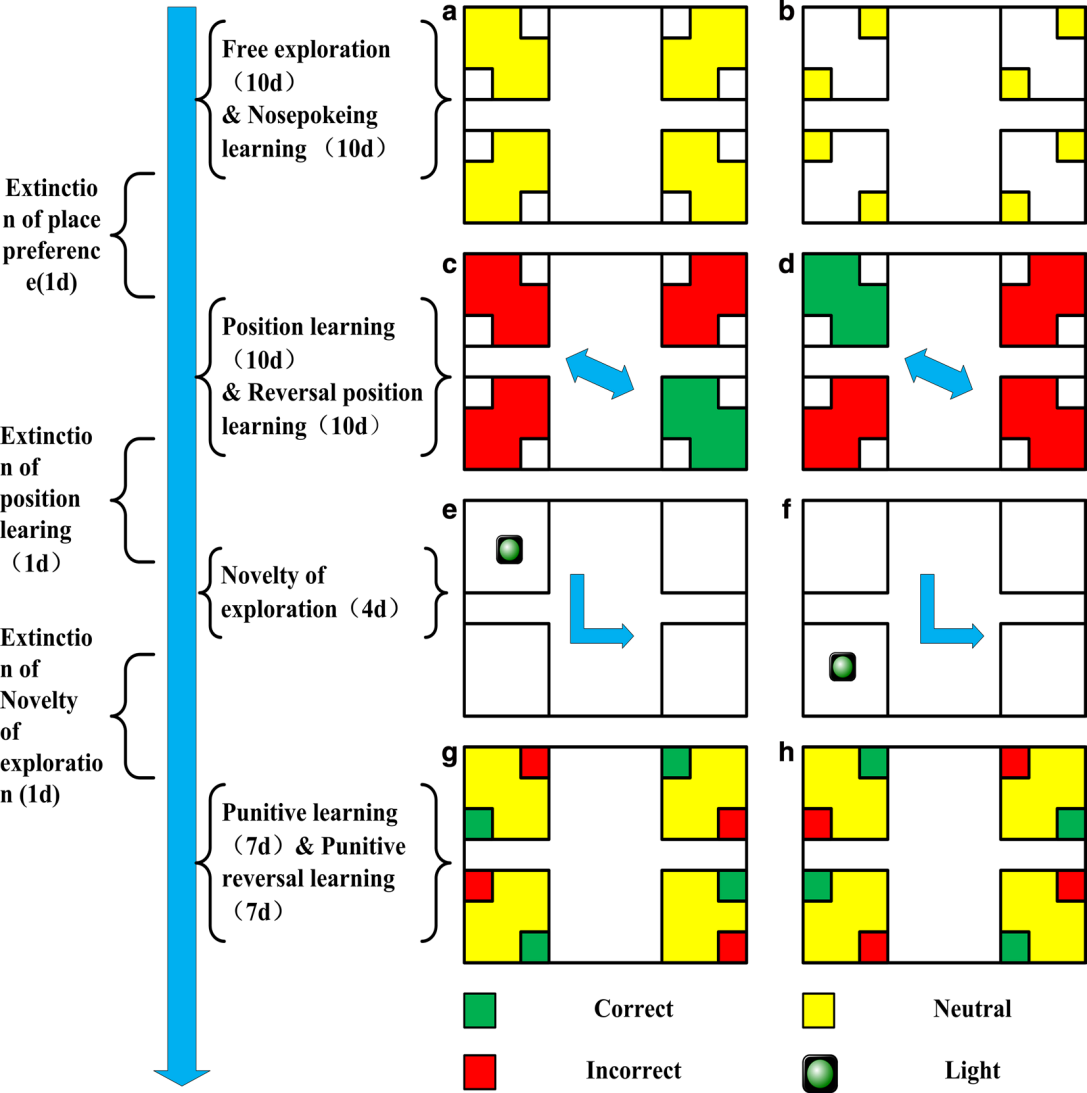
Intellicage learning module design: **a** free exploration test. **b** Nosepoke learning test. **c** Positional learning ability test. **d** Position reversal learning ability test **e**, **f** novel things to explore the test. **g** Punitive learning test. **h** Punitive reversal ability test

Two weeks after surgery, rats were transferred to IntelliCages. Learning and memory information were collected from 9:00 a.m. to 12:00 a.m. Drug perfusion was performed half an hour before the experiment. At the end of each trial, rats were removed, fed freely in a single cage and water-cut (7:00 a.m.–9:00 a.m.) (Fig. 1).

Intellicage learning module design:

1. *Free exploration* free exploration to allow rats to become, familiar with the environment for 10 days. All doors can be opened to reach the water bottle. The number of corner visits and nosepokes was monitored to assess rat exploratory power and corner preferences.
2. *Nosepoke learning* for a total of 10 days, all doors were closed and rats must complete the nosepoke to open the door to drink water. The number of corner visits and nosepokes was monitored to assess rat exploratory power and corner preferences. Special attention was directed to determine the least preferred corner of each rat in order to guide the next module design.
3. *Behavioural extinguishment* rats were allowed to explore for 1 day to extinguish the previous learning behaviour, during which time all rats were able to access all corners and vial vents.
4. *Position learning* for a total of 10 days, the rat’s least preferred corner of the nosepoke adaptation period was designated as “correct”, while the remaining corners were designated as “error”. All rats were able to visit all the corners, but only when the corner was “correct”, would the door could be opened and drinking allowed. The position learning ability was measured by calculating the number of correct corner visits.
5. *Position reversal learning* for a total of 10 days, the opposite corner of the “correct” corner in the position learning was designated as the new “correct” corner and remaining corners were designated as “error”. Rats were able to enter all places freely, The position reversal learning ability was measured by calculating the number of correct corner visits.
6. *Behavioural extinguishment* the same as in step3.
7. *Novelty exploration* for a total of 4 days, the LED lights of one corner were opened randomly. The light shifted in a counter-clockwise direction on each day. All rats were free to enter all corners, the number of corners visited was calculated, and the “novelty object preference” was evaluated.
8. *Behavioural extinguishment* the same as in step3.
9. *Punitive learning* for 7 days, the leftside of all the corners was assigned as its “correct” (reward) side. Unlike position learning, in this module, rat nosepoke to the “error” side incurred a blow penalty (aversion to irritation).
10. Penalty reversal learning: for 7 days, the rightside of all the corners was assigned as the “correct” (reward) side and nosepoke of the “error” side was penalized.

### Sample collection

After behavioural tests, the rats were sacrificed following anaesthesia with 10% chloral hydrate and the insular tissues of some rats were collected. The other rats were perfused with 4% Paraformaldehyde solution. The isolated brains were stored in sucrose solution to dehydrate gradiently and embedded in OCT for frozen tissue sections.

### Immunofluorescence

The immunofluorescence method of SABC (StreptAvidin Biotin Complex) staining was performed as below. After antigen retrieval using citric acid buffer, the sections were blocked with serum and incubated with antibodies (GB_1_, 1:300; GB_2_, 1:500) at 4 °C overnight. After washing in PBS, donkeyanti-rabbit-FITC (green) fluorescence second antibody drop, room temperature 1 h, PBS wash 10 min × 4 times. The cartridge was dried and directly blocked with anti-quencher. The insular GI areas of immunostained slides were observed and positive cells were quantitatively analyzed using Image J1.48 analysis system. Six fields of view were picked for every slide.

### RT-PCR

Total RNA of insular tissues was extracted using TRIzol reagent according to the manufacturer’s protocol. First-strand cDNA was generated using M-MLV reverse transcriptase. PCR was performed to detect the mRNA expression of each gene. PCR application conditions were described as followed: denaturation at 94 °C for 3 min, followed by 40 cycles of denaturation at 94 °C for 30 s, annealing at 58 °C for 30 s and extension at 72 °C for 45 s. RT-PCR products were analyzed and visualized on 4% agarose gel containing ethidium bromide (EB). Images were captured by Tanon 3500 digital gel imaging system. The PCR primers used were listed in Table 1.

**Table 1:** Specific primers used in real-time PCR analysis

Gene	Primer	Sequence (5′→3′)
GAPDH	FW RV	GAGTCAACGGATTTGGTCGT GACAAGATTCCCGTTCTCAG
GB1	FW RV	AGATTGTGGACCCCTTGCAC AGAAAATGCCAAGCCACGTA
GB2	FW RV	CACCGAGTGTGACAATGCAAA CCAGATTCCAGCCTTGGAGG

### Western blotting

Insular tissues were lysed with protein extraction kit on ice, and then total protein content was determined by BCA protein determination method. Protein from each sample was separated by SDS/PAGE and transfer on to a PVDF membrane. After blocking with 5% non-fat milk for 1 h, the membranes were immunoblotted with primary antibodies (GB_1_, 1:300; GB_2_, 1:500) overnight. After incubated with secondary antibody (1:5000), signal detection was performed by Odyssey infrared laser imaging system, followed by gray intensity analysis.

### Statistical analysis

All results are expressed as the mean ± SD. Analysis was performed using SPSS 21.0 software. For the behaviour test, the significance of the difference between groups was measured using one-way ANOVA followed by Tukey test for comparison between two groups. For RT-PCR and Western blotting, the significance of the difference between groups was measured using one-way ANOVA followed by Bonferroni’s Multiple Comparison test for comparison between two groups*. P* < 0.05 was considered to be statistically significant (*P < 0.05, **P < 0.01).

## Results

### Behavioural test

#### Intellicage

**Fig. 2:**
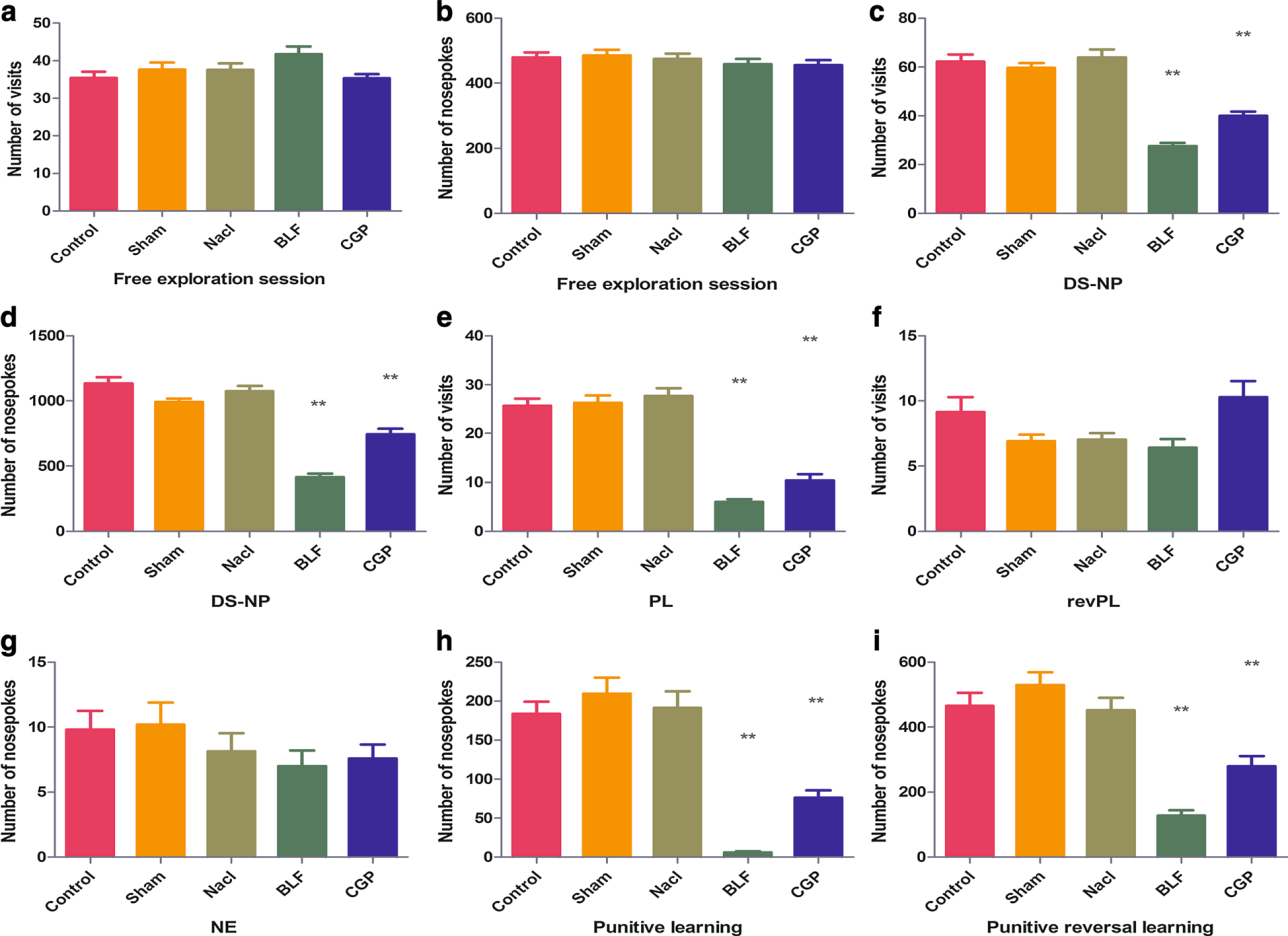
Behavioural test (**a**). **b** Free exploratory: there was no significant difference in visit and nosepoke between the five groups of rats, indicating that the model preparation had no effect on the basic learning ability of rats. **c** Number of visits in nosepoke learning: In the BLF and CGP groups, the number of visits decreased (*P* < 0.01, compared with the other three groups), and the BLF group decreased significantly (*P* < 0.01, compared with the CGP group). **d** Number of nosepokes in nosepoke learning: The BLF and CGP groups showed decreased numbers of nosepokes (*P* < 0.01, compared with the other three groups). The decreasing trend in the BLF group was more obvious (*P* < 0.01, compared with the CGP group). **e** Number of correct visits in position learning: the number of correct visits decreased in the BLF and CGP groups (*P* < 0.01, compared with the other three groups). **f** Number of correct visits in position reversal learning: There was no statistically significant reduction in the number of correct visits in the BLF and CPG groups compared with the other three groups (*P* < 0.01). **g** Number of visits in novelty exploration: The results showed no significant difference in the visiting times of five groups of rats. **h** Number of correct nosepokes in punitive learning: The BLF and CGP groups exhibited fewer correct nosepokes (*P* < 0.05, compared with the other three control groups), and the number in the BLF group was significantly lower (*P* < 0.05, compared with the CGP group). **i** Number of correct nosepokes in punitive reversal learning: The correct number of nosepokes in the BLF and CGP groups was reduced (*P* < 0.01, compared with the other three groups), and the BLF group had the least number of correct nosepokes (*P* < 0.05, compared with the CGP group)

Free exploration: There was no statistically significant difference in terms of the visit [F (4, 245) = 2.272, *P* > 0.05] (Fig. 2a) and nosepoke [F (4, 245) = 0.693, *P* > 0.05] (Fig. 2b) measures of learning ability between the five groups of rats. The results showed that under normal conditions, the cognitive learning ability of the five groups of rats was basically the same.

#### Nosepoke learning

In terms of the number of visits and nosepokes, compared with the other three groups, the visit rate of the BLF and CGP groups was decreased [F (4, 245) = 48.12, *P* < 0.01]. Furthermore, the number of visits in the BLF group was significantly reduced compared with the CGP group [q = 5.385, *P* < 0.01] (Fig. 2c). This finding suggests that baclofen and CGP35348 can inhibit spatial learning in normal rats, but the baclofen inhibitory effect is stronger. Additionally, the number of nosepokes in the BLF group was significantly lower than in the CGP group [F (4, 245) = 57.15, *P* < 0.01], [q = 8.44, *P* < 0.01] (Fig. 2d). This finding indicates that baclofen and CGP35348 also inhibit the skills of learning ability of normal rats and that inhibition by baclofen was stronger.

#### Position learning

The correct number of visits of the BLF and CGP groups was significantly lower than those of the other three groups [F (4, 245) = 56.26, *P* < 0.01], while there was no significant difference between the BLF group and the CGP group (q = 3.164, *P* > *0.05*) (Fig. 2e). This indicates that baclofen and CGP35348 can inhibit spatial learning and memory in normal rats.

#### Position reversal learning

There were no significant differences in the number of correct visits between the BLF and the CGP groups compared with the other three groups, but the number of correct visits for the BLF group was significantly lower than that of the CGP group [q = 4.45, *P* < *0.05*] (Fig. 2f). This suggests that baclofen and CGP35348 have an effect on learning and memory and that baclofen has a stronger inhibitory effect on the reversal of spatial position learning, which may not affect the spatial learning ability of rats.

#### Novelty exploration

The learning ability of five groups of rats did not show statistical significance in terms of the number of visits [F (4, 95) = 1.039, *P* > 0.05] (Fig. 2g). The results showed that for the five groups of rats the novelty exploration of learning ability was basically the same.

#### Punitive learning

The number of correct nosepokes in the BLF group and the CGP group was significantly lower than that in the other three groups [F (4, 170) = 32.20, *P* < 0.01], and the BLF and CGP groups showed a statistically significant difference in nosepokes [q = 4.48, *P* < 0.05] (Fig. 2h). This result indicates that baclofen and CGP35348 could inhibit the spatial and skills learning components of recognition memory in normal rats and that the inhibitory effect of baclofen was stronger.

#### Punitive reversal learning

The number of correct nosepokes in the BLF and CGP groups was significantly lower than that in the other three groups [F (4, 170) = 23.01, *P* < 0.01], while the BLF and CGP groups showed a statistically significant difference in terms of the number of correct nosepokes [q = 4.46, *P* < 0.05] (Fig. 2i). This finding indicates that baclofen and CGP35348 had inhibitory effects on spatial and skills learning of recognition memory and that baclofen had a stronger inhibitory effect.

The conversion efficiency of learning and memory:

1. During the first 5 days of position reversal learning, the BLF group showed less frequent visits compared with the other four groups [F (4, 245) = 5.611, *P* < 0.01]. In addition, the number of correct visits decreased significantly in the BLF group compared with the CGP group [q = 4.14, *P* < 0.01] (Fig. 3a). However, in the second 5 days, the BLF and CGP groups showed the same performance as the control groups in terms of the number of correct visits. The number of correct visits in the BLF group was significantly lower than that in the CGP group [q = 3.95, *P* < 0.01] (Fig. 3b), which indicates that, at the beginning of reversal learning, baclofen inhibits the conversion efficiency of learning more than CGP35348. The response ability of rats to spatial localization transformation is poor, but after a period of learning, Baclofen’s inhibition slowly weakens.

**Fig. 3:**
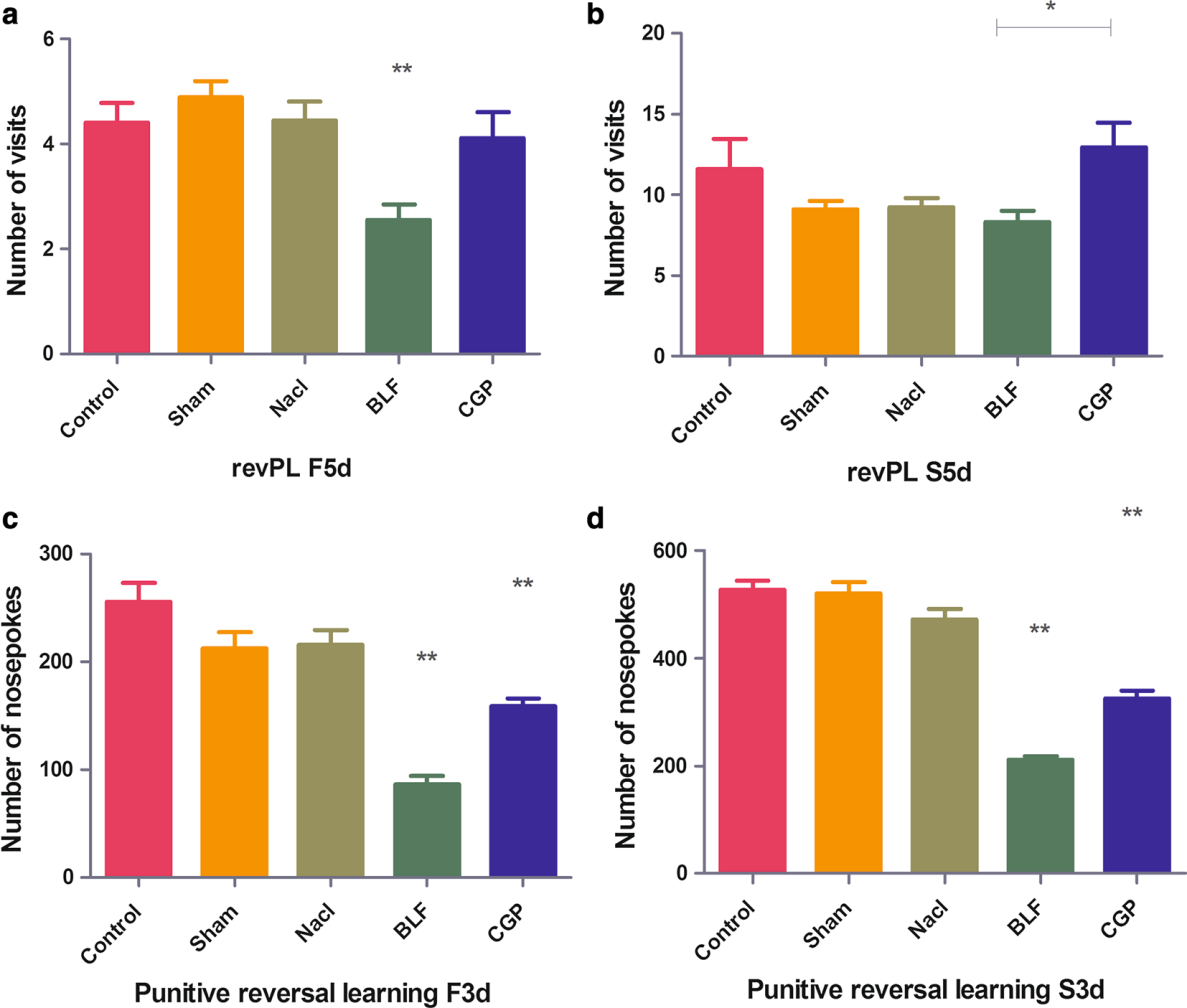
Reversing learning efficiency change of position reversal learning and penetration reversal learning. **a**, **b** Position reversal learning: reversing learning efficiency change of position reversal learning: in the BLF group, the learning efficiency was significantly inhibited in the first 5 days (*P* < 0.05, compared with the CGP group). When the CGP group was compared with the other three groups, the difference in learning efficiency was not statistically significant. In the BLF group, learning efficiency was inhibited in the second 5 days (*P* < 0.05), but there was no significant difference in learning efficiency compared with the other three control groups. The results of the CGP group were the same as those of the BLF group. **c**, **d** Penetration reversal learning: reversing learning efficiency change of penetration reversal learning: Learning efficiency was inhibited in the BLF and CGP groups in the first 3 days, and the difference was statistically significant (*P* < 0.01, compared with the other groups). The learning ability of the animals in the BLF group was worse (*P* < 0.01, compared with the CGP group). The learning efficiency was significantly inhibited in the BLF and CGP groups in the last 3 days, the difference was statistically significant (*P* < 0.01, compared with the other groups), and the learning ability of the BLF group worsened (*P* < 0.01 compared with the CGP group)
2. In the BLF and CGP groups, the number of correct nosepokes decreased significantly from the first 3 days [F (4, 70) = 25.56, *P* < 0.01] to the last 3 days of the reversal of learning [F (4, 70) = 68.17, *P* < 0.01]. The BLF and CGP groups differed significantly from each other [q = 5.56, *P* < 0.01] (Fig. 3c), [q = 6.76, *P* < 0.01] (Fig. 3d). This finding indicated that baclofen and CGP35348 could inhibit the learning efficiency of rats. Baclofen produced a more obvious reduction in the reversal of learning efficiency.

### Nissl

**Fig. 4:**
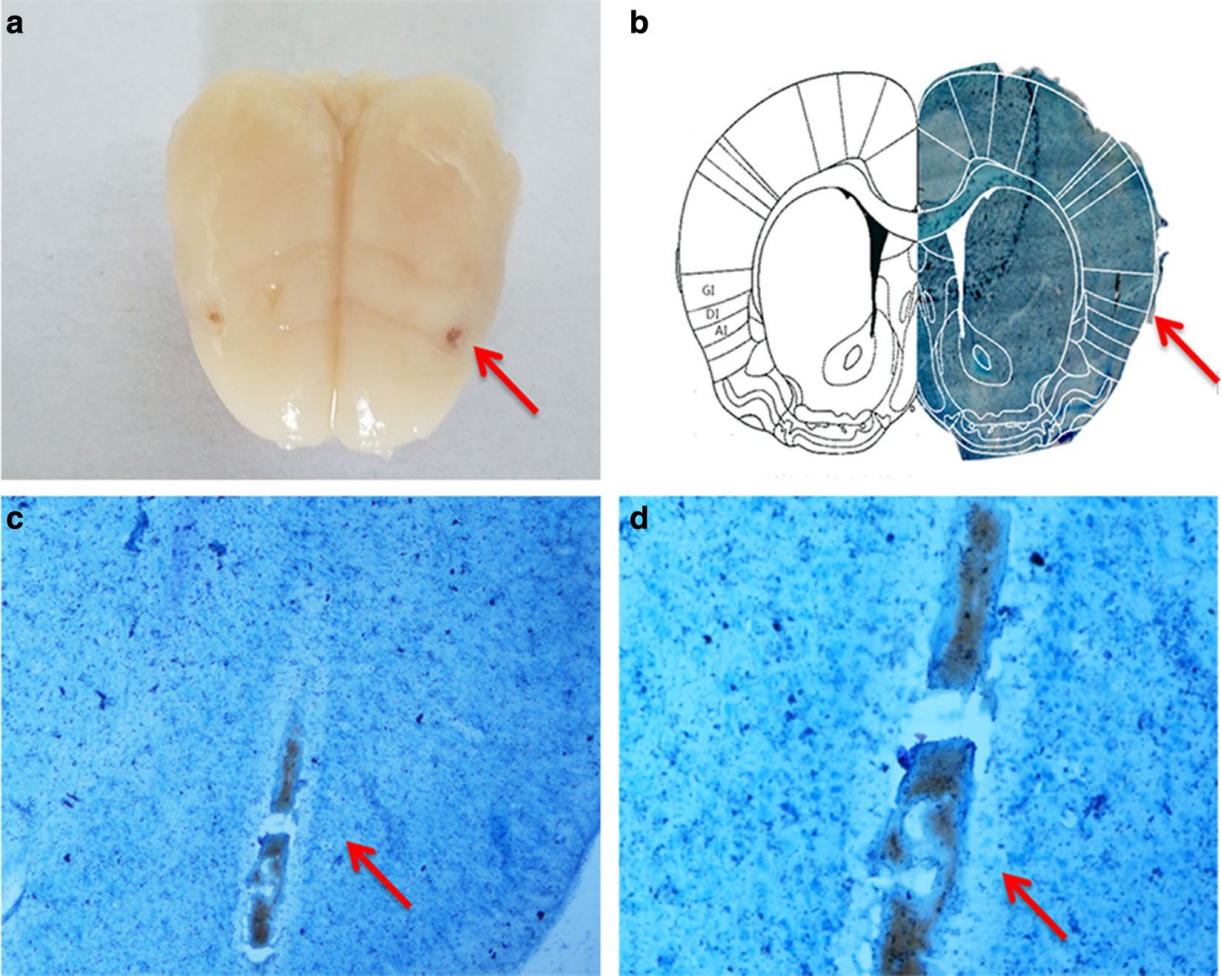
Nissl (**a**). The appearance of bilateral insular inserts (**b**). Comparison of position and mapping of the left lobes. *Right* a Nissl-stained frozen section of the rat brain (coronal cut, +1.2 mm relative to bregma [39]) was microinjected into the granular insular cortex as detailed in "Methods". *Left* a scheme of the corresponding contralateral hemisphere. *GI* granular insular cortex, *DI* dysgranular insular cortex. **c** ×200 target position of insular, **d** ×400 target position of insular

Nissl staining showed that the target of the insula was in accordance with the experimental requirement (Fig. 4).

### Immunofluorescence

**Fig. 5:**
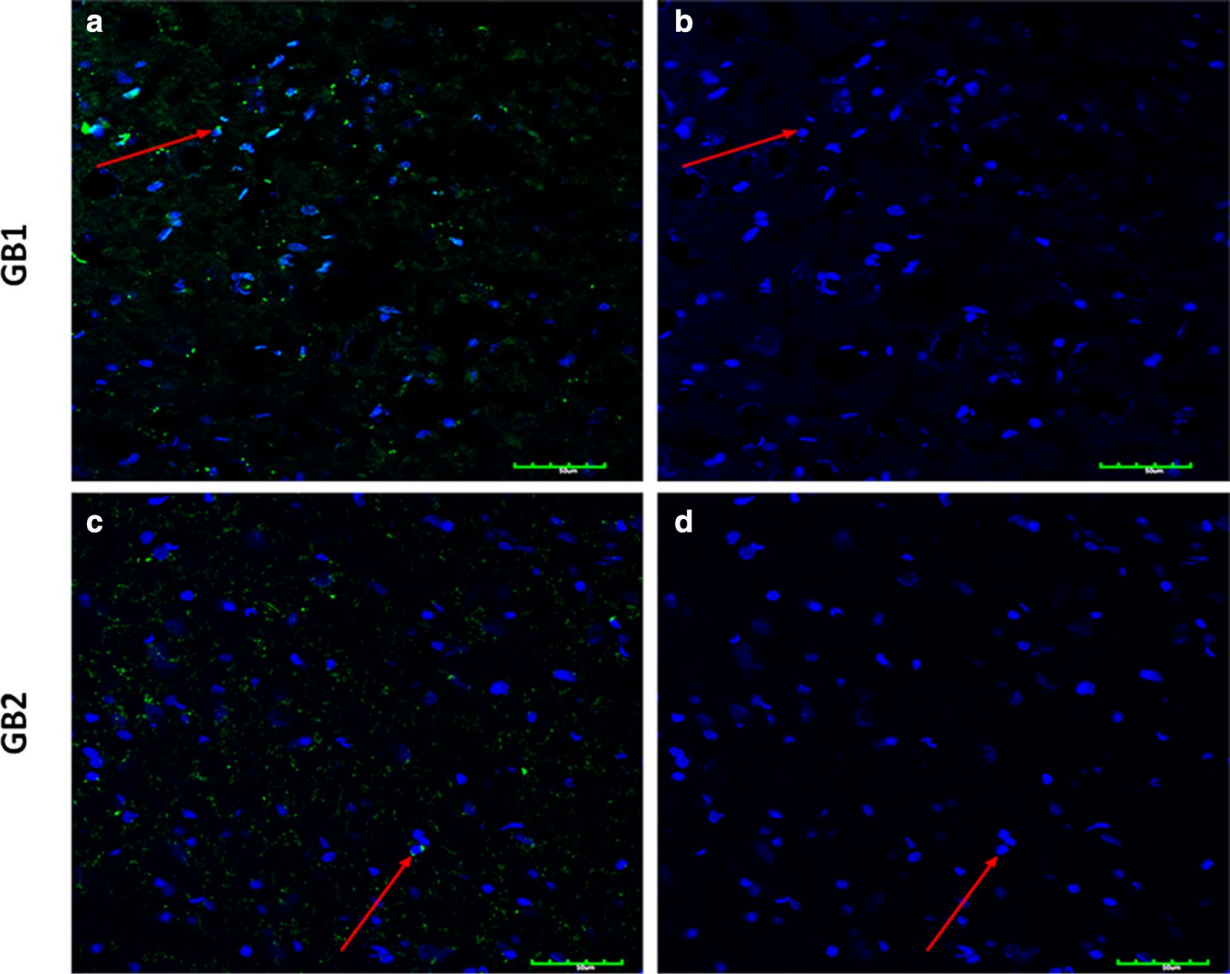
Immunofluorescence: Immunofluorescence of GB1 and GB2 specific markers in insula of the control group. *Scale bars* 25 mm (GB1) and 50 mm (GB2). **a** GB1 expression FITC (*green*), **b** DAPI, **c** GB2 expression FITC (*green*), **d** DAPI

Immunofluorescence showed normal expression of GB1 and GB2 in the insula, indicating that the normal rat insula contains GABA_B_R (Fig. 5).

### RT-PCR

**Fig. 6:**
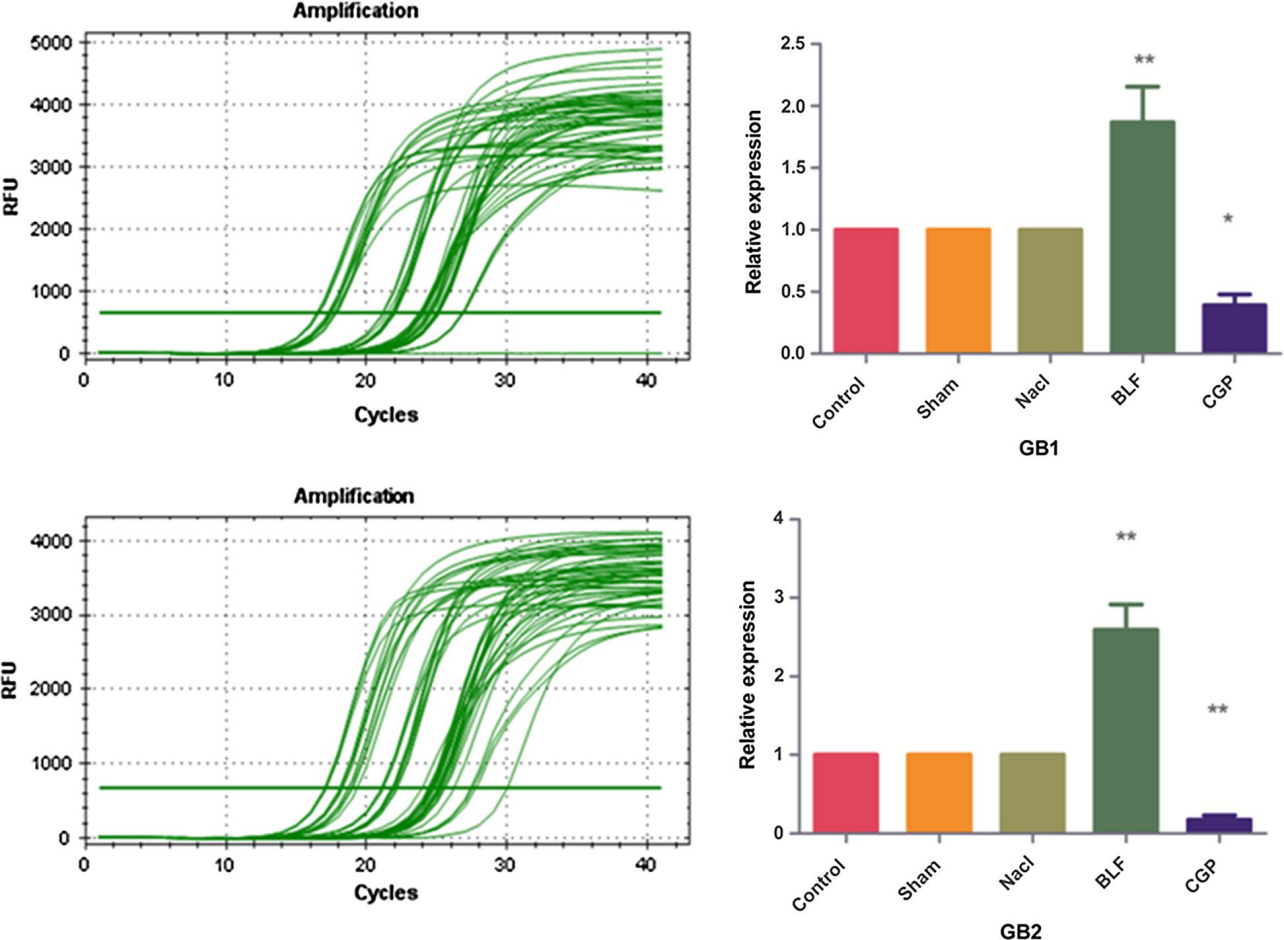
Relative expression of GB_1_ and GB_2_ at mRNA level, GB_1_ and GB_2_ were upregulated in the Baclofen group and downregulated in the CGP group (*P* < 0.01)

The expression of GB1and GB2 was much higher in the Baclofen group but lower in the CGP group compared to the other groups [GB1F (4, 20) = 15.51, *P* < 0.01] [GB2F (4, 20) = 35.98, *P* < 0.01] (Fig. 6).

### Western blotting

**Fig. 7:**
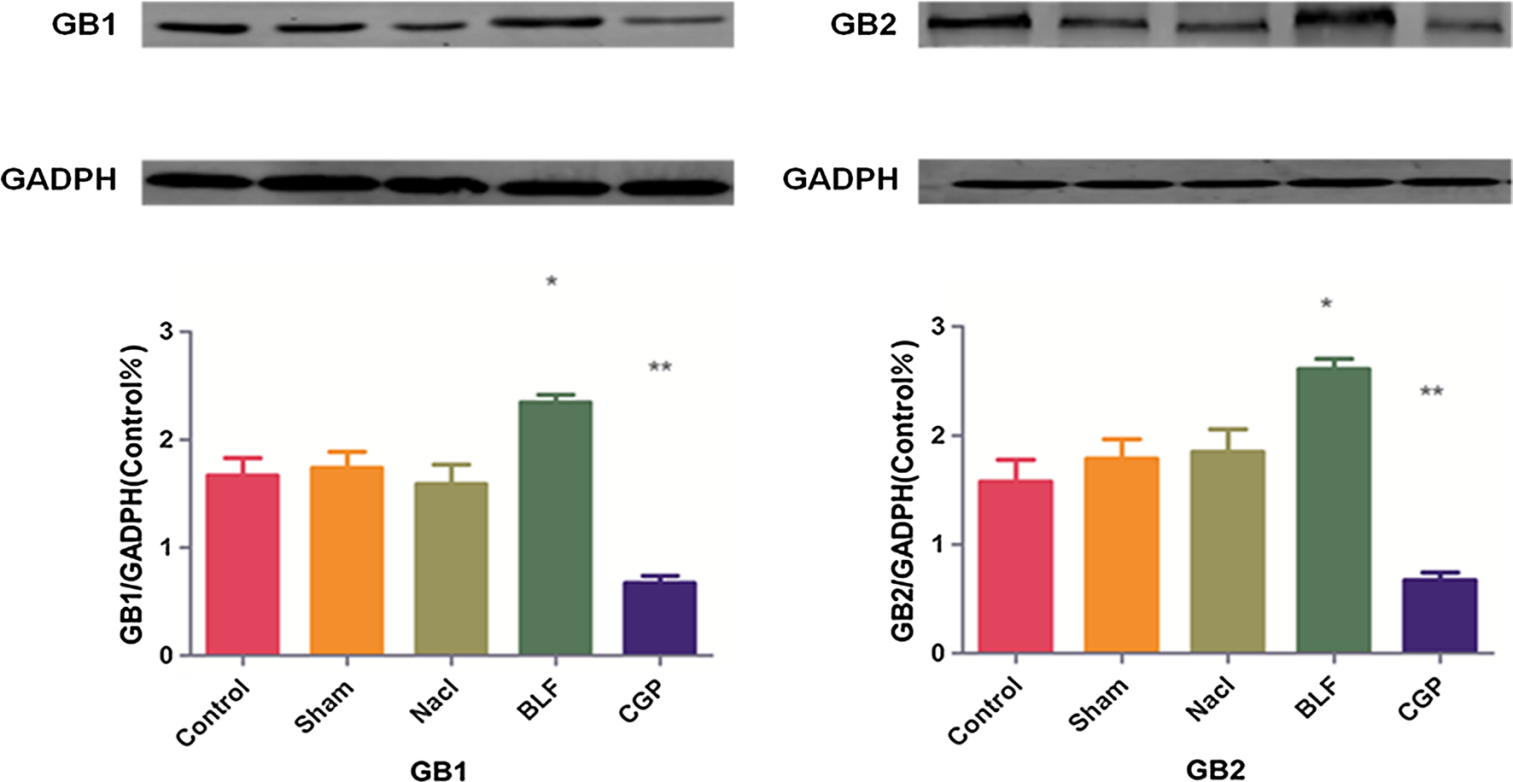
Western blotting: For the five groups, relative expression of GB1 and GB2. At the protein level expression increased in the Baclofen group but decreased in the CGP group significantly. *P < 0.05, **P < 0.01

The expression of GB1 and GB2 was much higher in the Baclofen group, but lower in the CGP group compared to the other groups [GB1F (4, 20) = 20.17, *P* < 0.01] [GB2F (4, 20) = 19.01, *P* < 0.01] (Fig. 7).

## Discussion

Research regarding the insula is a new method of understanding the cognitive function of the brain. Studies have shown that the insula is involved in the regulation of pain, formation of addiction, formation of disgust, generation of depression, regulation of cardiac activity, language planning, and empathy [1, 2, 23]. Herpes encephalitis, ischaemic stroke, glioma and other diseases often involve the insula. In addition, neurological and psychiatric disorders, such as temporal lobe epilepsy, schizophrenia, dementia, Alzheimer’s disease, anxiety, depression, and autism, are closely related to the insula [24]. Some research on recognition memory has shown that the main structural basis of recognition memory from the perspective of anatomy and function is the highlight network. However, the role and mechanism of the insula, which is the core of the highlight network, is unclear. In addition, there is still considerable controversy regarding the relationship between recollection and familiar memory, its various functional characteristics, the neural basis and other issues [19, 24]. However, GABA, as the most important inhibitory neurotransmitter in the central nervous system, plays an important role in the encoding, sorting and transmission of nerve information [25]. Several studies have shown that GABA is involved in the decision-making process [26] and that the dysfunction of GABA_B_R is also associated with a variety of neurological diseases, including epilepsy, anxiety, depression, drug addiction and cognitive disorders [11, 27–29]. To summarize the results of the research regarding GABA and the insula, first, neuropsychiatric diseases are closely associated to the insula and GABA_B_R. Second, neuropsychiatric diseases are often accompanied by impaired cognitive function. Third, the recognition of cognitive memory is a good indicator of cognitive function. As both the insula and GABA_B_R play important roles in recognition memory, we raise some questions of whether there is a link between them. This question was explored in our experiments.

Our behavioural testing utilized a new intelligent behaviour monitoring system, intellicage, with different recognition memory modules. This system is different from the previous water maze, dark test and other classical behavioural detection methods. One of the biggest drawbacks of these classic behavioural experiments is that they are man-made to provide animals a variety of stimuli to observe the animal’s learning and memory changes. Alternatively, intellicage is characterized by the location of rats in an environment that closely resembles a natural social environment. This setup avoids human intervention as much as possible, which can lead to behavioural changes in rats [30, 31]. After years of use, intellicage is gradually being taken seriously in the field of behavioural research. Using the intellicage operating system, we divided the testing programme into four modules, which represent four different types of recognition memory, to verify the effect of GABA_B_R expression in the insula on recognition memory. First, we used nosepoke learning to explore the effects of rats’ recognition memory (skills learning ability). The second module used position learning and position reversal learning to explore rats’ spatial recognition memory changes. In the third module, we explored the use of novel exploration to evaluate the ability of rats to recognize memories of novel things. The fourth module utilized punitive learning and punitive reversal learning to explore a reconsidering memory that reflected spatial positioning and skills learning ability.

Our evidence confirms that after perfusing the GABA_B_R-selective agonist baclofen and GABA_B_R-specific antagonist CGP35348 in normal rat insula, the number of corner accesses was reduced in the nosepoke study. This finding indicated that GABA_B_R upregulation and downregulation in the insula resulted in a decrease in the skills learning ability associated with recognition memory. Studies have confirmed that the anterior medial temporal lobes plays a role in food intake [32], and our results confirmed that GABA_B_R in the insula also affects skills learning as rats learn to drink and remember how to obtain water. In position learning, modulation of rat GABA_B_R expression also caused a reduction in spatial memory, but the change in GABA_B_R expression did not affect the spatial recognition memory of the rat in position reversal learning. This finding suggests that upregulation and downregulation of GABA_B_R in the insula could damage the rat’s spatial memory, but it did not affect spatial reversal learning associated with recognition memory. Studies have confirmed that the hippocampus is the node of the memory system [33], but we have determined through experiments that GABA_B_R in the insula also has such a role. For punitive learning and punitive reversal learning, upregulation and downregulation of GABA_B_R expression in the insula of rats could lead to a decrease in the number of correct nosepokes, which indicates that the comprehensive utilization of recognition memory is impaired and interrupted. In recent years, most studies on recognition memory have targeted the hippocampus [34], It has been reported that in the hippocampus, baclofen inhibits GABA_B_R-induced spatial learning in normal rats by activating the TREK-2K^+^ channel [15]. CGP35348 inhibits the inhibitory postsynaptic potential (IPSP), enhances GABA_B_R activation, and improves the memory formation process [35]. However, in our study, up-regulation and down-regulation of GABA_B_R expression in the insula during the abovementioned test modules affected cognition in normal rats, and GABA_B_R upregulation in the normal rat insula was more damaging to cognition. Therefore, it cannot be determined whether CGP35348 can improve cognitive function because there are different targets and the roles of the targets are also very different. Thus, increases or decreases in GABA_B_R expression in the insula will damage memory function. Moreover, with a change in learning pattern, the cognitive changes associated with GABA_B_R expression were different. Several studies have confirmed that GABA in the hippocampus plays an important role in novelty recognition and target identification [36, 37], but we found that the change in GABA_B_R expression in the insula did not affect the ability of the animals to recognize new things, which indicates that the insula may not be the region involved in novelty recognition.

In this experiment, we also explored the conversion efficiency of rats’ recognition memory. In position reversal learning, we found that in the first 5 days, upregulation and downregulation of GABA_B_R in the insula resulted in a marked decrease in the recognition memory of rats and that upregulation of GABA_B_R expression made this reduction more obvious. However, in the last 5 days of learning, GABA_B_R expression in the insula was not associated with a decline in recognition memory capacity. This finding indicates that at the beginning of the first 5 days, upregulation and downregulation of GABA_B_R expression in the insula resulted in a reduction in the efficiency of rat spatial recognition of memory transfer, but with an increase in learning time, the efficiency of this conversion was restored in the last 5 days. However, upregulation and downregulation of GABA_B_R expression, which occurred both in the first 3 days and in the second 3 days of punitive reversal learning, impaired the conversion efficiency of spatial location recognition memory and the recognition memory of the skills learning ability, suggesting that changes in GABA_B_R expression in the insula influence the efficiency of the transformation of recognition memory. Combining the results of position reversal learning and punitive reversal learning, we conclude that GABA_B_R changes in the insula can lead to a reduction in the efficiency of recognition memory, which is a single mode, and this reduction in efficiency will be mitigated with increased learning time; however, for more than one mode of recognition memory, the change in GABA_B_R expression in the insula will continue to affect the conversion efficiency of recognition memory.

The insula is located near the temporal lobe. Research has confirmed that the medial temporal lobe and insula are associated with memory consolidation [6] and that temporal lobe lesions, such as temporal lobe epilepsy, can cause patients to have memory damage [38]. It has been found that the relationship between GABA_B_R expression in the insula and memory recognition may provide a new target for the treatment of patients with impaired cognitive function. However, in future experiments, we need to study the link between the insula and other brain regions to investigate the behavioural changes of rats in a large sample and to explore the molecular mechanism of GABA_B_R expression in insular and recognition memory.

In conclusion, our study demonstrates that GABA_B_R plays an important role in the formation of recognition memory and may be become a new target for the study of memory.

## Abbreviations

GABA: gamma-aminobutyric acid
PL: position learning
revPL: position reversal learning
NE: novelty of exploration
F5d: five days before
S5d: the second five days
F3d: three days before
S3d: the last three days
